# Extramammary Paget’s disease affecting the external auditory canal: a case report

**DOI:** 10.1186/1752-1947-7-250

**Published:** 2013-11-07

**Authors:** Francisco de Assis Castro Bomfim, Walber de Oliveira Mendes, Igor Moreira Veras, Jônatas Catunda de Freitas, Ana Maria da Silva Castro, Francisco Monteiro de Castro

**Affiliations:** 1 Department of Head and Neck Surgery, Walter Cantídio Hospital, 608 Prof. Costa Mendes Street, Rodolfo Teófilo, Fortaleza, Ceará ZIP Code: 60430-140, Brazil; 2 Head and Neck Surgery Research Group, Faculty of Medicine, Federal University of Ceará, 608 Prof. Costa Mendes Street, Rodolfo Teófilo, Fortaleza, Ceará ZIP Code: 60430-140, Brazil; 3 Radiotherapy Department, Regional Center for Integrated Oncology (CRIO), 1300 Francisco Calaça Street, Fortaleza, Ceará ZIP Code: 60336-550, Brazil; 4 Livino Pinheiro Laboratory, Haroldo Juaçaba Hospital, Cancer Institute of Ceará (ICC), 1222 Papi Junior Street, Fortaleza, Ceará ZIP Code: 60430-230, Brazil

**Keywords:** External auditory canal, External ear, Extramammary, Head-neck tumors, Paget’s disease

## Abstract

**Introduction:**

Extramammary Paget’s disease is a rare histological type of intraepithelial adenocarcinoma that mainly affects apocrine sweat gland-rich areas. Predilection sites include the anogenital region and, less commonly, the axillae. These tumors rarely occur in non-apocrine regions. The aim of this case report is to describe a case of extramammary Paget’s disease in the external auditory canal with extensive temporal bone involvement and skull base invasion.

**Case presentation:**

A 40-year-old Caucasian man presented with a progressively growing, vegetating lesion localized in his right-hand external auditory canal. He had peripheral facial nerve paralysis and complete ipsilateral hearing loss. An incisional biopsy suggested extramammary Paget’s disease, and the immunohistochemical analysis confirmed the diagnosis. The tumor was considered inoperable because of the extensive skull base involvement, and he was referred for palliative radiotherapy.

**Conclusion:**

We report here the third case of extramammary Paget’s disease affecting the external auditory canal to be described in the literature. In all the three cases, the prognosis was unfavorable despite treatment.

## Introduction

Extramammary Paget’s disease (EMPD) is a rare histological type of intraepithelial adenocarcinoma that mainly affects apocrine sweat gland-rich areas. The predilection sites include the anogenital region and, less commonly, the axillae [1]. These tumors rarely occur in non-apocrine regions. Despite the presence of modified ceruminous sweat glands in the external auditory canal, there have been only two previously reported cases of EMPD in this region [2,3]. The aim of this case report is to describe the case of a patient with EMPD in the external auditory canal, with extensive temporal bone involvement and skull base invasion. The patient had a good initial response to radiotherapy but developed a lung metastasis and died 4 months after the end of treatment.

## Case presentation

**Figure 1:**
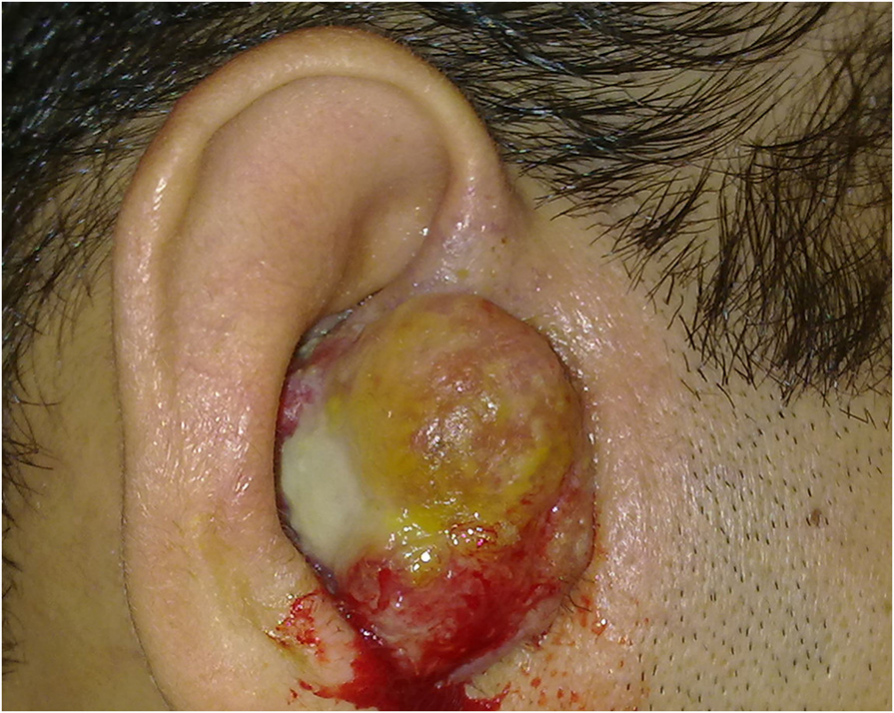
Clinical features of the patient with extramammary Paget’s disease.

The patient was a 40-year-old Caucasian man who presented with a 3-month history of a progressively growing, vegetating lesion localized in his right-hand external auditory canal. Upon physical examination, a 2.5cm bleeding, ulcerated lesion was observed to occlude his right external auditory canal (Figure 1). The patient had already developed peripheral facial nerve paralysis and complete ipsilateral hearing loss.

**Figure 2:**
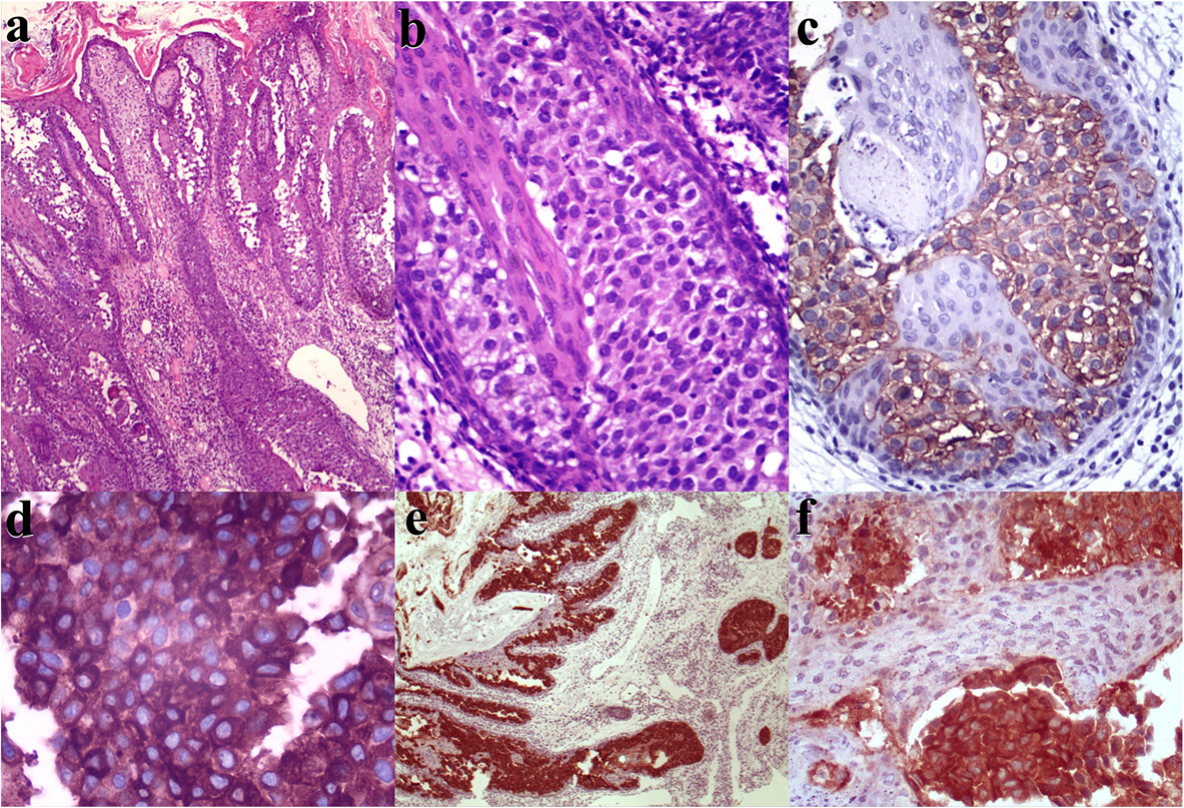
**Histopathological examination and immunohistochemical study.** Detailed legend: **(a)** An extensive infiltrate with involvement largely concentrated in the deep epidermis. Epidermal hyperplasia and hyperkeratosis occurred to some degree (hematoxylin and eosin staining, original magnification ×40). **(b)** The tumor cells were large, with distinct borders, abundant pale cytoplasm, pleomorphic nuclei, and conspicuous nucleoli (hematoxylin and eosin staining, original magnification ×200). **(c)** Strong human epidermal growth factor receptor 2-positive membranous reactivity was detected in Paget’s cells in the epidermis (original magnification ×200). **(d)** Paget’s cells showing diffuse positivity for carcinoembryonic antigen (original magnification ×400). **(e)** Extramammary Paget’s disease manifested as cytokeratin-positive malignant cell epidermal infiltration (original magnification ×40). **(f)** Reactivity for gross cystic disease fluid protein of 15kDa (original magnification ×200).

**Figure 3:**
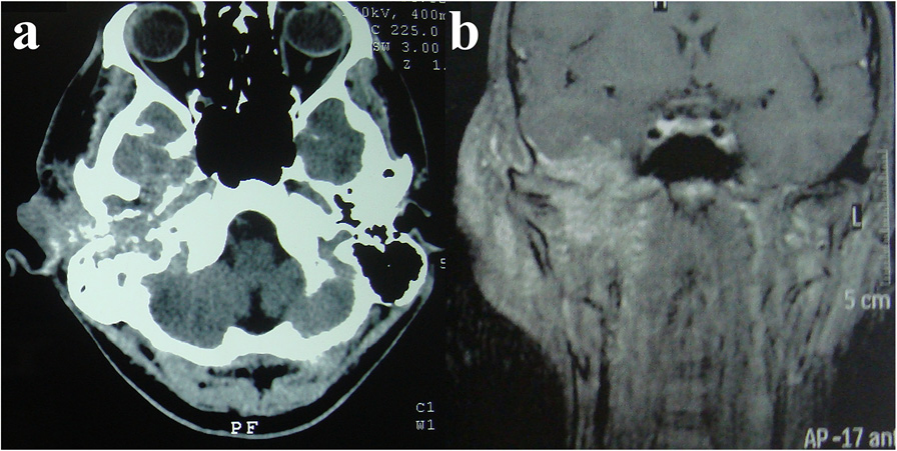
**Computed tomography and magnetic resonance imaging.** Detailed legend: Computed tomography axial image **(a)** and magnetic resonance imaging coronal image **(b)** of the head, performed without **(a)** and with **(b)** the administration of contrast material, showing the infiltrative lesion in the external right ear and its relation to anatomical structures.

The incisional biopsy suggested EMPD (Figure 2a, b), but there was a possible differential diagnosis of amelanotic melanoma or pagetoid Bowen’s disease. The immunohistochemical study (Table 1) was positive for human epidermal growth factor receptor 2 (Her2 or Neu; Figure 2c), carcinoembryonic antigen (CEA; Figure 2d), cytokeratin-7 (CK7; Figure 2e), and gross cystic disease fluid protein of 15kDa (GCDFP-15; Figure 2f), confirming the diagnosis of EMPD. A computed tomography scan revealed an infiltrative lesion in his external right ear, with paravertebral and middle ear extensions that affected his internal carotid artery anterior wall and facial nerve, respectively (Figure 3a). Magnetic resonance imaging revealed an expansive tumor with imprecise limits and no cleavage plane with the carotid artery. An extension to the parapharyngeal space and an invasion of the middle fossa, associated with meningeal contrast enhancement, were observed (Figure 3b).

**Table 1:** Immunohistochemical analysis

**Antibody**	**Antibody dilution**	**Result**
p63	1/300	Negative
CK7	1/100	Positive (+++)
HMB-45	1/400	Negative
Melan-A	1/400	Negative
34BE12	1/200	Negative
Cerb-B2	1/1500	Positive (+++)
CEA mono	1/200	Focally positive (++)
CEA poly	1/2000	Positive (+++)
GCDFP-15	1/50	Positive (++)

*Abbreviations: CEA mono* carcinoembryonic antigen monoclonal antibody, *CEA poly* CEA polyclonal antibody, *CK7* cytokeratin-7, *GCDFP-15* gross cystic disease fluid protein of 15kDa, *HMB-45* human melanoma black-45, *Melan-A* melanoma antigen.

The tumor was considered inoperable because of the extensive skull base involvement, and the patient was referred for palliative radiotherapy. The patient underwent 36 sessions of radiotherapy and was given a total radiation dose of 72Gy. A complete response was achieved at the primary site, but during treatment, diffuse bilateral pulmonary metastases were detected. The patient experienced significant weight loss during treatment and died 4 months after completing radiotherapy.

## Discussion

EMPD was first reported by Crocker in 1889 [4] and is a rare type of adenocarcinoma of the apocrine sweat glands with intraepithelial extension [1] that primarily affects sites with high densities of these glands, such as the genital, perianal, and axillary regions [5]. Other sites are quite rarely affected; these include the external auditory canal [2,3], upper eyelid (glands of Moll) [6], and umbilical region [7], where there is evidence of modified apocrine glands. In addition, EMPD can occur in places with no evidence of apocrine glands, such as the face [8]. In such cases, EMPD is described as ectopic.

In its usual presentation in the genital and perianal regions, EMPD is reported as a slow-growing, erythematous, and well-demarcated lesion that forms crusts, scaly edges, and occasional ulcerations. These tumors grow until achieving local invasion and metastasis [9]. Precise diagnoses are often delayed for years due to nonspecific appearances [10]. The behaviors of EMPD that have been described in the literature are very different from those observed in the present case, in which the lesion presented with rapid and aggressive growth, including lymph node metastasis within a few months, and led to the patient’s death.

Cases of EMPD that affect the external auditory canal are extremely rare. Such a case was first published by Kaneko and Fligiel [3] in 1975 and then by Gonzalez-Castro *et al*. [2] in 1998. In both cases, EMPD was very aggressive, and the patients died within months of the diagnoses, despite treatment. The case described here is the third reported case. Adenocarcinomas that arise from the ceruminous glands of the external auditory canal are rare malignancies that usually present as growing and painful masses in the external auditory canals of middle-aged patients [11]. Besides ear pain and rapid tumor growth, the most common symptoms include hearing impairments such as hearing loss, deafness and tinnitus, discharge, infection, bleeding, facial nerve paralysis, paresthesia, dizziness, and balance disorders [12].

Immunohistochemistry is critical for diagnosis because the histological aspect can resemble amelanotic melanoma or pagetoid Bowen’s disease [8]. Markers such as CEA and low-molecular-weight cytokeratins, including PKK1, CK7, GR53, and 35 Beta H11, are often positive in Paget’s cells and apocrine sweat glands [13]. In 2009, Plaza *et al*. [14] published a study on a series of 47 patients with EMPD, in which more than 30% expressed the Her2 (Neu) receptor. These cases were associated with potentially more aggressive lesions and had higher rates of recurrence after treatment. The patient in the present case had tumor cells positive for CEA, CK7, Her2 (Neu), and GCDFP-15.

Combined surgical resection and adjuvant radiotherapy provides the most effective treatment [1]; however, several series showed good results with radiotherapy alone in inoperable cases [8]. For metastatic EMPD, most authors have recommended combinations of multiple chemotherapy drugs such as 5-fluorouracil, mitomycin C, vincristine, cisplatin, and epirubicin in combination with radiotherapy [15]. However, the serious adverse effects of these treatment regimens should be taken into account [13]. The detection of Her2 (Neu) in a subgroup of patients with EMPD could provide a new starting point for immunotherapy in a manner similar to therapy for Her2 (Neu)-positive breast cancer patients [13,14].

The prognosis of EMPD in its usual presentation is generally favorable and depends on the extent of the lesion, depth of invasion, and lymph node spread [10]. The associated mortality rate ranges from 13% to 18% [13], and the survival rate at 5 years is 72% [10].

## Conclusions

In summary, EMPD very rarely affects the external auditory canal, and only two cases have been previously described in the literature. In this case and the two previous cases, the prognosis was unfavorable despite treatment. If the patient had sought medical attention earlier, the disease could possibly have been diagnosed at an initial stage and treated with radical surgery combined with adjuvant radiotherapy and could possibly have been healed.

## Consent

Written informed consent was obtained from the patient’s next of kin for publication of this case report and the accompanying images. A copy of the written consent is available for review by the Editor-in-Chief of this journal.

## Competing interests

The authors declare that they have no competing interests.

## Authors’ contributions

FACBJ cared for the patient and analyzed and interpreted the patient data. WOM wrote the manuscript and was solely responsible for the literature search. IMV performed radiotherapy planning and reviewed the radiotherapy details. AMSC performed the histopathological examination and immunohistochemical study. JCF and FMCJ were involved in the revision of the manuscript. All the authors read and approved the final manuscript.
